# Obesity and recurrence‐free survival in patients with hepatocellular carcinoma after achieving sustained virological response to interferon therapy for chronic hepatitis C

**DOI:** 10.1002/ags3.12183

**Published:** 2018-06-22

**Authors:** Hiroji Shinkawa, Shogo Tanaka, Shigekazu Takemura, Tokuji Ito, Takanori Aota, Masaki Koda, Toru Miyazaki, Takatsugu Yamamoto, Shoji Kubo

**Affiliations:** ^1^ Department of Hepato‐Biliary‐Pancreatic Surgery Osaka City University Graduate School of Medicine Osaka Japan; ^2^ Department of Surgery Asakayama General Hospital Sakai Japan

**Keywords:** hepatocellular carcinoma, interferon therapy, obesity, recurrence, sustained virological response

## Abstract

**Aim:**

Some patients who achieve a sustained virological response (SVR) to interferon (IFN) treatment for chronic hepatitis C prior to hepatic resection for hepatocellular carcinoma (HCC) experience postoperative recurrence. This study investigated the relationship between obesity and postoperative HCC recurrence in SVR patients.

**Methods:**

Fifty‐nine patients who had achieved SVR before hepatic resection were evaluated. Patients had a solitary tumor ≤5 cm in diameter or ≤3 lesions each ≤3 cm in size with no macroscopic vascular invasion (Milan criteria). Patient characteristics potentially associated with recurrence risk were investigated.

**Results:**

Three‐, 5‐, and 7‐year recurrence‐free survival after surgery were 65%, 44%, and 41%, respectively. Univariate analysis showed that obesity (*P* < .01), hypertension (*P* = .038), and non‐anatomical resection (*P* = .022) were significantly associated with a lower recurrence‐free survival rate. In a multivariate analysis, obesity (hazard ratio, 2.8; 95% confidence interval [CI] 1.3‐6.1; *P* < .01) and non‐anatomical resection (hazard ratio, 2.7; 95% CI 1.1‐6.2; *P* = .025) were independently associated with postoperative recurrence. Three‐, 5‐, and 7‐year overall survival rates after surgery were 100%, 80%, and 64% in obese patients and 100%, 92%, and 82% in non‐obese patients, respectively (*P* = .014). However, other variables showed no significant difference in the overall survival rate.

**Conclusions:**

Obesity and non‐anatomical resection were independent risk factors for HCC recurrence after hepatic resection and successful IFN therapy. Obesity is an important clinical problem to consider to improve postoperative outcomes in such patients.

## 1 INTRODUCTION

Hepatocellular carcinoma (HCC) is the sixth most common cancer worldwide, with an increasing incidence in Western countries,^1^ and chronic hepatitis C virus (HCV) infection is one of the causes. Interferon (IFN) can reduce hepatic inflammation and fibrosis and reduce the risk of HCC recurrence after resection of HCV‐related HCC, especially in patients with a sustained virological response (SVR).^2^ However, the annual incidence of HCC recurrence after HCV eradication is 0.3%‐2.4%.^3^, ^4^ The recognized risk factors for HCC development after SVR include advanced age, hepatic fibrosis, and alcohol abuse.^4^, ^5^ However, the risk factors associated with HCC recurrence after hepatic resection in SVR patients are unclear.

Obesity is a serious public health problem worldwide, and its incidence is increasing in Japan along with the Westernization of lifestyles. Obesity is closely associated with the development of metabolic syndrome, which includes hypertension, cardiovascular disease, and diabetes mellitus, and is independently associated with the risk of developing several malignancies, including breast, renal, and pancreatic cancer.^6^ Obesity has also been reported to be independently associated with the risk of hepatocarcinogenesis^6^, ^7^ resulting from the accumulation of fat in the liver and consequent hepatocellular injury.^6^, ^8^, ^9^ Obesity may influence HCC recurrence, but its involvement in postoperative recurrence in SVR patients with hepatic resection for HCC has not been investigated.

In the present study, we estimated the impact of obesity on HCC recurrence after hepatic resection following successful IFN therapy for HCV infection.

## 2 MATERIALS AND METHODS

### 2.1 Patients

A total of 68 patients who were seronegative for hepatitis B surface antigen and seropositive for anti‐HCV antibodies but had an SVR and a history of curative hepatic resection for HCC between January 1994 and December 2015 at the Department of Hepato‐Biliary‐Pancreatic Surgery, Osaka City University Hospital, were eligible. SVR was defined as a serum HCV‐RNA under the detection sensitivity limit at 6 months after the termination of IFN therapy.

Of the 68 eligible patients, we excluded nine with HCC outside the Milan criteria, which specify a solitary tumor ≤5 cm in diameter or ≤3 lesions, each ≤3 cm in diameter, without macroscopic vascular invasion. Tumors exceeding the Milan criteria are considered advanced and to have a potentially increased risk of recurrence. Curative hepatic resection was defined as complete resection of recognizable tumors and the histological absence of tumor cells along the parenchymal transection line.

Of the 59 remaining enrolled patients, 27 had received IFN monotherapy (IFN‐α in 19, IFN‐β in 1, and IFN alpha‐2b in 7). Five patients had received IFN alpha‐2b/ribavirin combination therapy, six had received pegylated (PEG)‐IFN monotherapy (PEG‐IFN alpha‐2a in three and PEG‐IFN alpha‐2b in three), and 21 had received PEG‐IFN/ribavirin combination therapy (PEG‐IFN alpha‐2a in three and PEG‐IFN alpha‐2b in 18). HCV strains were identified by genotyping in 36 patients and by serotyping in 21. The HCV strains in two patients were unidentified, as the test was not available at the regional hospital.

The study was conducted following the guidelines of the Ethics Committee of our institution and the Declaration of Helsinki and approved by the Ethics Committee of Osaka City University (No. 3815). Informed consent was obtained from each patient.

### 2.2 Metabolic syndrome‐related factors

The metabolic syndrome‐related factors assessed were obesity, diabetes mellitus, dyslipidemia, and a history of hypertension. Obesity was defined as a body mass index (BMI) ≥25 kg/m^2^ following the criterion for adult Asian populations.^10^ Diabetes mellitus was defined as a fasting serum glucose ≥126 mg/dL or the use of antidiabetes medications.^11^ Dyslipidemia was defined as serum triglycerides ≥150 mg/dL or the use of indicated medications.^12^ A history of hypertension was determined by interview. To evaluate the change in BMI after surgery, body weight measured at 3, 6, or 12 months after surgery was carried out.

### 2.3 Surgical treatment

Indication for surgery was based on an algorithm consisting of the presence/absence of ascites, serum total bilirubin level, and results of the indocyanine green (ICG) retention test.^13^ For anatomical resection, we divided the hepatic parenchyma along the demarcation line appearing after the occlusion of the portal vein and hepatic artery in hemihepatectomy and sectoriectomy, or after the injection of ICG into the portal vein conflicting the tumor‐bearing area in segmentectomy. In non‐anatomical resection, we divided the hepatic parenchyma along the line to maintain a surgical margin of 5 mm.

### 2.4 Detection of recurrence

Patients were evaluated by serum alpha‐fetoprotein, ultrasonography, computed tomography, or magnetic resonance imaging 1 month after surgery and every 3 months thereafter. If recurrence of HCC was suspected, selective hepatic angiography and an ultrasound‐guided biopsy were used to establish a definitive diagnosis, as required. Sites of recurrence were categorized into near the resection site (NR), which was the residual part of the tumor‐bearing third‐order portal branches and the part adjacent to the liver transection surface, or distant from the resection site (DR).

### 2.5 Histology

Histological evaluation followed the guidelines of the Liver Cancer Study Group of Japan.^14^ Grade (severity of active hepatitis) and stage (degree of hepatic fibrosis) of non‐malignant hepatic tissue were scored using a histological activity index^15^, ^16^ including periportal necrosis with or without bridging necrosis, intralobular degeneration with focal necrosis, portal inflammation, and fibrosis. Non‐alcoholic fatty liver disease activity score, which concentrates on macrovesicular steatosis, lobular inflammation, and hepatocyte ballooning injury, was used to assess the degree of hepatic steatosis and inflammation. Details of the three components of the score are as follows: steatosis grade (<5% = 0, 5%‐33% = 1, 33%‐66% = 2, and >66% = 3); lobular inflammation (no foci = 0, <2 foci per 200× field = 1, 2‐4 foci per 200× field = 2, >4 foci per 200× field = 3); and hepatocyte ballooning (none = 0, few balloon cells = 1, many cells/prominent ballooning = 2).^17^

### 2.6 Statistical analyses

Differences in clinicopathological findings were tested with the Mann‐Whitney *U*‐test and Fisher's exact probability method. Kaplan‐Meier method was used to calculate recurrence‐free survival rates, and between‐group differences in the survival were evaluated by log‐rank test. Cox's proportional hazard model was used for multivariate analysis.

Variables potentially associated with recurrence were selected based on previous study results or on our own clinical experience^5^, ^8^ and included age (<65 or ≥65 years); gender; history of alcohol abuse (i.e. intake of at least 86 g of ethanol daily for at least 10 years); hepatitis B core (HBc) antibody positivity; interval from the end of IFN therapy to the detection of HCC (≤5 or >5 years); alanine aminotransferase (ALT) activity (≤30 or >30 IU/L); total bilirubin (T‐Bil) level (≤1.0 or >1.0 mg/dL); albumin concentration (≥4.0 or <4.0 g/dL); platelet count (>15 × 10^4^ or ≤15 × 10^4^/mL); serum alpha‐fetoprotein (AFP) (≤20 or >20 ng/mL); largest diameter of the main tumor (>2.0 or ≤2.0 cm); single or multiple tumors, including intrahepatic metastases; microscopic vascular invasion; degree of differentiation of the main tumor (well‐differentiated, moderate, or poor), grading (0‐2 or 3‐4); staging (0‐3 or 4; liver cirrhosis); steatosis (negative 0 or positive 1‐3); lobular inflammation (negative 0/1 or positive 2/3); ballooning (negative 0 or positive 1/2); and type of hepatic resection (i.e. anatomical resection, including hemihepatectomy, sectoriectomy, or segmentectomy or limited non‐anatomical resection).

Influence of metabolic syndrome‐related variables and clinical and pathological characteristics on recurrence‐free survival was analyzed. *P*‐values were derived from two‐tailed tests, and the threshold for statistical significance was set at *P* < .05.

## 3 RESULTS

### 3.1 Patient characteristics

Clinicopathological characteristics of the 59 patients are shown in Table S1. Mean age was 66 years (range, 48‐76 years), 48 were men and 11 women, 31% were obese, 29% had diabetes mellitus, 20% had dyslipidemia, and 44% had hypertension. Twenty‐seven patients had hepatic steatosis scores of 0, 17 had scores of 1, 11 had scores of 2, and four had scores of 3.

### 3.2 Survival outcomes

**Figure 1:**
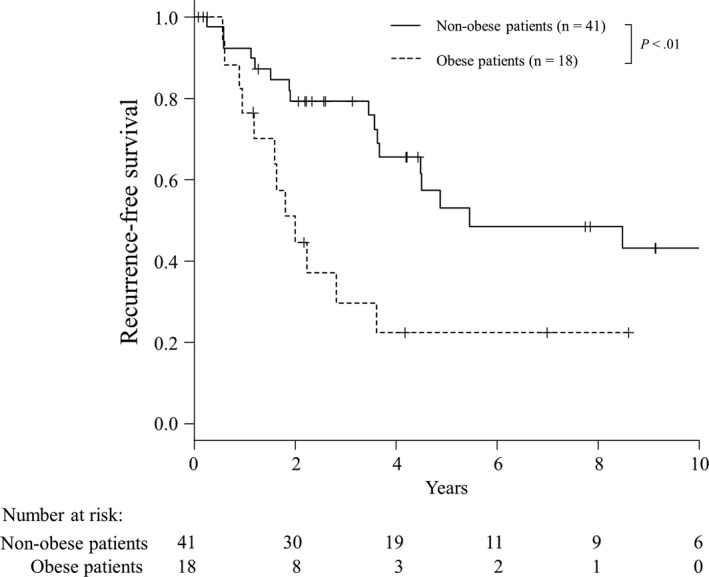
Recurrence‐free survival rates after hepatic resection of hepatocellular carcinoma in obese (n = 18) and non‐obese patients (n = 41)

The 3‐, 5‐, and 7‐year recurrence‐free survival rates after surgery were 65%, 44%, and 41%, respectively. Twenty‐nine patients (49%) experienced recurrence, all of which were in the remnant liver. Univariate analysis showed that obesity (*P* < .01), hypertension (*P* = .038), and non‐anatomical resection (*P* = .022) were significantly associated with a lower recurrence‐free survival rate (Table 1). Recurrence‐free survival in obese and non‐obese patients is shown in Figure 1. Multivariate analysis found that obesity (hazard ratio, 2.8; 95% confidence interval [CI] 1.3‐6.1; *P* < .01) and non‐anatomic resection (hazard ratio, 2.7; 95% CI 1.1‐6.2; *P* = .025) were independent risk factors for postoperative recurrence (Table 2).

**Table 1:** Recurrence‐free survival after surgery in patients with chronic hepatitis C

Variable	Number	MST (days)	Survival rate (years)			*P*‐value
			3	5	7	
Age (years)						
<65	21	3102	67	51	51	.72
≥65	38	1644	64	41	36	
Gender						
Female	11	1639	80	50	50	.60
Male	48	1339	61	42	38	
Alcohol abuse						
Presence	13	1319	53	35	23	.26
Absence	46	1784	69	47	47	
Diabetes mellitus						
Presence	17	1319	59	49	33	.44
Absence	42	1644	67	43	43	
Obesity						
Presence	18	731	30	22	22	<.01
Absence	41	1996	75	54	49	
Dyslipidemia						
Presence	12	NA	81	65	65	.22
Absence	47	1339	61	40	36	
Hypertension						
Presence	26	1303	50	35	28	.038
Absence	33	3102	76	51	51	
HBc antibody						
Positive	33	1639	74	43	43	.61
Negative	26	1784	52	46	39	
Interval from IFN^a^ (years)						
≤5	39	1324	59	38	38	.22
>5	20	1996	77	55	47	
T‐Bil (mg/dL)						
<1.0	48	1339	60	41	38	.49
≥1.0	11	3102	82	53	53	
Albumin (g/dL)						
<4.0	14	1324	63	45	45	.88
≥4.0	45	1644	65	44	40	
Platelet count (×10^4^/mL)						
<15	29	1319	62	41	35	.30
≥15	30	1784	68	48	48	
ALT (IU/L)						
>30	21	1784	68	46	46	.42
≤30	38	1639	63	42	37	
α‐Fetoprotein (ng/mL)						
>20	17	1784	55	46	46	.83
≤20	42	1639	69	43	38	
Tumor size (cm)						
>2.0	29	1303	54	45	45	.93
≤2.0	30	1644	75	38	30	
Differentiation degree^b^						
Well, mod	45	1644	67	45	40	.72
Poor	14	1263	56	40	40	
No. tumors						
Single	50	1784	67	48	44	.075
Multiple	9	945	50	17	17	
MVI						
Presence	18	3102	59	59	59	>.99
Absence	41	1639	67	38	33	
Hepatic steatosis						
Positive	32	1319	56	43	37	.32
Negative	27	1784	75	46	46	
Lobular inflammation						
Positive	47	NA	62	41	41	.37
Negative	12	1324	75	64	54	
Ballooning						
Positive	24	1324	59	39	31	.33
Negative	35	1644	69	47	47	
Liver cirrhosis						
Presence	15	1028	44	44	44	.37
Absence	44	1644	72	44	40	
Type of hepatic resection						
Non‐anatomical	37	1303	51	25	25	.022
Anatomical	22	NA	79	66	60	

a Interval from the end of interferon therapy to the detection of hepatocellular carcinoma.

b Tumor differentiation: well, well‐differentiated; mod, moderately differentiated; poor, poorly differentiated.

ALT, alanine aminotransferase; HBc, hepatitis B core; IFN, interferon; MST, median survival time; MVI, microvascular invasion; NA, not applicable; T‐Bil, total bilirubin.

**Table 2:** Multivariate analysis of the patient characteristics associated with postoperative recurrence

	Multivariate risk ratio^a^	*P*‐value
Obesity	2.8 (1.3‐6.1)	<.01
Non‐anatomical resection	2.7 (1.1‐6.2)	.025

a Risk ratio (95% confidence interval).

The 3‐, 5‐, and 7‐year overall survival rates after surgery were 100%, 89%, and 77%, respectively. A total of 11 patients died during the study period, nine as a result of cancer‐related causes and two from other causes. Among obese patients, all five patients died of cancer‐related causes. The 3‐, 5‐, and 7‐year overall survival rates after surgery were 100%, 80%, and 64% in obese patients and 100%, 92%, and 82% in non‐obese patients, respectively (*P* = .014). Other variables showed no significant difference in the overall survival rate (Table S2).

### 3.3 Characteristics of obese and non‐obese patients

Obesity was an independent risk factor for recurrence in this patient series (Table 3). Comparison of the clinicopathological characteristics of obese and non‐obese patients showed that age, gender ratio, history of alcohol abuse, presence of diabetes mellitus, tumor characteristics, and type of surgery in obese and non‐obese patients were not significantly different. However, the prevalence of hepatic steatosis was significantly higher in obese patients (72%) than in non‐obese patients (56%) (*P* = .023). Furthermore, we evaluated the change in BMI after surgery in obese and non‐obese patients. Of 59 patients, 50 patients had body weight measurements taken at 3, 6, or 12 months after surgery without recurrence. In obese patients (n = 17), median pre‐ and postoperative BMI were 27.2 (range, 25.2‐32.5 kg/m^2^) and 26.8 (range, 24.4‐33.3 kg/m^2^), respectively. (*P* = .58) In non‐obese patients (n = 33), median pre‐ and postoperative BMI were 22.6 (range, 18.7‐24.8 kg/m^2^) and 23 (range, 18.6‐25.4 kg/m^2^), respectively. (*P* = .93) No significant difference in BMI change after surgery was confirmed in obese and non‐obese patients. Among obese patients, improvement of obesity (from BMI ≥25 kg/m^2^ to BMI < 25 kg/m^2^) was observed in four patients. Median recurrence‐free and overall survival times were 26.8 months and 85.9 months in patients with improvement of obesity (n = 4), and 19.6 months and 84.7 months in patients without improvement of obesity (n = 13), respectively.

**Table 3:** Characteristics of obese and non‐obese patients with chronic hepatitis C

Variable	Obese patients (n = 18)	Non‐obese patients (n = 41)	*P*‐value
Gender (male/female)	15/3	33/8	>.99
Age (years)			
≥65 (n = 38)	14	24	.24
<65 (n = 21)	4	17	
Alcohol abuse (+/−)	6/12	7/34	.19
Diabetes mellitus (+/−)	8/10	9/32	.12
Dyslipidemia (+/−)	2/16	10/31	.31
Hypertension (+/−)	10/8	16/25	.27
HBc antibody (+/−)	7/11	26/15	.096
Interval from IFN^a^ (years)			
≤5	10	29	.37
>5	8	12	
Total bilirubin (mg/dL)			
<1.0 (n = 48)	14	34	.72
≥1.0 (n = 11)	4	7	
Albumin (g/dL)			
<4.0 (n = 12)	5	9	.74
≥4.0 (n = 40)	13	32	
Platelet count (×10^4^/mL)			
<15 (n = 29)	12	17	.095
≥15 (n = 30)	6	24	
ALT (IU/L)			
>30 (n = 21)	7	14	.77
≤30 (n = 38)	11	27	
α‐Fetoprotein (ng/mL)			
>20 (n = 17)	7	10	.35
≤20 (n = 42)	11	31	
Tumor size (cm)			
>2.0 (n = 29)	11	18	.27
≤2.0 (n = 30)	7	23	
Differentiation degree (well, mod/poor)^b^	11/7	34/7	.098
Tumor no. (single/multiple)	16/2	34/7	.71
MVI (+/−)	5/13	13/28	>.99
Hepatic steatosis (+/−)	14/4	18/23	.023
Lobular inflammation (+/−)	16/2	31/10	.31
Ballooning (+/−)	9/9	15/26	.40
Grading score (0‐1 or 2‐4)	9/9	26/15	.40
Liver cirrhosis (+/−)	7/11	8/33	.19
Anatomical resection (+/−)	5/13	17/24	.39

a Interval from the end of interferon therapy to the detection of hepatocellular carcinoma.

b Tumor differentiation: well, well‐differentiated; mod, moderately differentiated; poor, poorly differentiated.

ALT, alanine aminotransferase; HBc, hepatitis B core; IFN, interferon; MVI, microvascular invasion.

### 3.4 Patterns of recurrence

Table 4 compares the prevalence of the site of recurrence and number of recurrent nodules between obese and non‐obese patients. In obese patients, NR and DR were identified in two patients (16.7%) and 10 patients (83.3%), respectively. In non‐obese patients, NR and DR were identified in five patients (29.4%) and 12 patients (70.6%), respectively. A solitary nodule of recurrence was observed in seven obese patients (58.3%) and in 13 non‐obese patients (76.5%). Furthermore, NR with multiple nodules was observed in one obese patient (8.3%) and in two non‐obese patients (11.8%), and DR with solitary nodule was observed in six obese patients (50.0%) and in seven non‐obese patients (41.2%).

**Table 4:** Recurrence patterns of obese and non‐obese patients

Characteristic	Obese patients (n = 18) (%)	Non‐obese patients (n = 41) (%)	*P*‐value
Recurrence	12 (66.7)	17 (41.5)	.095
Site of recurrence^a^			
Near resection site	2 (16.7)	5 (29.4)	.66
Distant from resection site	10 (83.3)	12 (70.6)	
No. of recurrent nodules^a^			
Solitary	7 (58.3)	13 (76.5)	.42
Multiple	5 (41.7)	4 (23.5)	
No. and site of recurrence^a^			
Multiple recurrences near resection site	1 (8.3)	2 (11.8)	>.99
Solitary recurrence distant from resection site	6 (50.0)	7 (41.2)	.72

a Percentage of all recurrences.

Table 5 compares the prevalence of the site of recurrence and number of recurrent nodules between patients with anatomical and non‐anatomical resections. All eight patients with anatomical resection experienced DR (100%). Among patients who received non‐anatomical resection, NR and DR were identified in seven patients (33.3%) and 14 patients (66.7%), respectively. A solitary nodule of recurrence was observed in seven anatomical patients (87.5%) and in 13 non‐anatomical patients (61.9%). NR with multiple nodules was observed only in three patients (14.3%) with non‐anatomical resection. DR with a solitary nodule was observed in seven anatomical patients (87.5%) and in nine non‐anatomical patients (42.9%).

**Table 5:** Recurrence patterns of anatomical and non‐anatomical resection

Characteristic	Anatomical resection (n = 22) (%)	Non‐anatomical resection (n = 37) (%)	*P*‐value
Recurrence	8 (36.4)	21 (56.8)	.18
Site of recurrence^a^			
Near resection site	0 (0)	7 (33.3)	.14
Distant from resection site	8 (100)	14 (66.7)	
No. of recurrent nodules^a^			
Solitary	7 (87.5)	13 (61.9)	.37
Multiple	1 (12.5)	8 (38.1)	
No. and site of recurrence^a^			
Multiple recurrences near resection site	0 (0)	3 (14.3)	>.99
Solitary recurrence distant from resection site	7 (87.5)	9 (42.9)	.044

a Percentage of all recurrences.

## 4 DISCUSSION

Multivariate analysis confirmed obesity and non‐anatomical resection as independent risk factors for postoperative HCC recurrence after successful IFN therapy, as the risk of recurrence was increased 2.8‐ and 2.7‐fold, respectively, in patients with these factors. To our knowledge, this is the first report of obesity as an independent risk factor for postoperative recurrence of HCC in patients with an SVR. An SVR might be expected to reduce the background hepatocarcinogenic potential in patients with a history of chronic hepatitis C and HCC surgery,^2^ as the tumor recurrence rate was 49%, and the recurrence‐free survival was 41% at 7 years after hepatic resection. These results indicate that the risk of HCC recurrence persists for a long time in patients who achieve an SVR.

Obesity may promote hepatocarcinogenesis.^6^, ^7^ In a large, population‐based study of more than 1.2 million men, a higher BMI in young adulthood was significantly associated with an increased risk of HCC.^18^ Ohki et al reported that a BMI >25 was an independent risk factor for HCC development in chronic hepatitis C patients, with a relative risk of 1.86.^8^ In this study, obese patients had a significantly increased risk of HCC recurrence. The cause of this increased risk is not clear, but adipokine‐related hepatocarcinogenesis might be involved. Obesity has been reported to affect hepatocarcinogenesis through adipose tissue remodeling with the secretion of proinflammatory adipokines and accumulation of ectopic lipids, resulting in liver lipotoxicity.^9^, ^19^ Level of serum adiponectin is inversely correlated with BMI, and a low serum adiponectin has been associated with an increase in the risk of various cancers.^8^, ^9^ Obesity may also promote hepatocarcinogenesis through hepatic steatosis, which can cause hepatic inflammation by a mechanism similar to that of non‐alcoholic steatohepatitis with pathological findings of lobular inflammation and hepatocellular injury (ballooning).^20^ However, in this study, hepatic steatosis, lobular inflammation, and ballooning were not independently associated with HCC recurrence. One possible reason for this discrepancy is the persistent influence of HCV infection. HCV infection can cause hepatic steatosis, and this steatosis persists even after HCV eradication.^3^ Another possible reason is the influence of alcohol abuse. Alcohol abuse is known to cause hepatic steatosis and steatohepatitis. These factors may have promoted hepatic steatosis, lobular inflammation, and ballooning in this study.

Hepatocellular carcinoma recurrence after curative treatment includes intrahepatic metastasis of original tumors and newly developed tumors (multicentric recurrence).^21^ Time to recurrence after surgery (early recurrence within 2 years of surgery or late recurrence at 2 or more years after surgery) is commonly used to distinguish the type of recurrence.^22^ In the present study, recurrence‐free survival showed that the difference in the recurrence rate between obese and non‐obese patients increased, even in the early post‐surgical period. However, on examining the patterns of recurrence between obese and non‐obese patients, multiple recurrences near the resection site were similarly observed between the two groups, whereas solitary recurrence distant from the resection site tended to be more frequently observed in obese patients than in non‐obese patients. Theoretically, recurrence derived from residual micrometastases can develop near the resection site. Furthermore, multiple recurrences near the resection site are considered likely to be a result of intrahepatic metastasis. In contrast, solitary intrahepatic recurrence distant from the resection site may be considered a pattern of multicentric recurrence.^22^ Therefore, multicentric recurrence may be associated with an increased risk of HCC recurrence in obese patients.

Non‐anatomical resection was also an independent risk factor for HCC recurrence. Anatomical resection along the portal tributaries is considered an effective way to remove occult intrahepatic HCC metastases.^23^, ^24^ In the present study, the patients who underwent anatomical resection had no multiple recurrences near the resection site. Furthermore, solitary recurrence distant from the resection site was more frequently observed in the patients who underwent anatomical resection than in those who underwent non‐anatomical resection. Therefore, recurrence derived from micrometastases of the original tumors might be markedly reduced by anatomical resection. We previously reported that anatomical resection might prevent HCC recurrence in patients achieving an SVR and a biochemical response (defined as normal ALT activity) for at least 1 year.^25^ Similarly, anatomical resection might be recommended for patients with HCC following HCV eradication.

In the present study, several common risk factors for HCC recurrence showed no significant difference between obese and non‐obese patients, including liver‐related factors^3^, ^4^ (albumin, ALT, T‐Bil, and liver cirrhosis) and tumor‐related factors^23^, ^25^ (AFP, microscopic vascular invasion, and tumor size). Furthermore, recurrence‐free survival rates tended to be more favorable in patients with albumin <4.0 g/dL, microscopic vascular invasion, tumor size >2.0 cm, AFP >20 ng/dL, ALT >30 IU/L, T‐Bil >1.0 mg/dL, and liver cirrhosis. One possible reason for these unexpected findings may be the small patient number. Another possible reason is the exclusion of patients with HCC outside the Milan criteria and the limitation of subjects to SVR patients. Patients who achieve an SVR typically have good liver function. Excluding advanced‐stage HCC patients and including only SVR patients may have reduced the recurrence risk associated with tumor progression and background liver disease in the present study. Positive reactivity of HBc antibody was also reported as a risk factor for HCC recurrence in HCV‐infected patients.^26^ However, positive reactivity of HBc antibody was not identified as a risk factor for recurrence in the present study. A previous report suggested that occult persistent HBV infection might contribute to hepatocarcinogenesis under conditions of continuous hepatitis caused by HCV.^26^ In patients with HCV eradication, the hepatocarcinogenic impact of HBc antibody positivity on the synergistic effects with HCV infection may be decreased. Furthermore, the hepatocarcinogenic effect of HBc antibody positivity itself can be obscured by tumor size, multiplicity, vascular invasion, and liver cirrhosis.^26^ Accumulation of more patients may clarify the influence of HBc antibody positivity on the risk of HCC recurrence in patients achieving SVR.

On comparing the overall survival, obese patients showed a more unfavorable outcome than non‐obese patients. Obesity is considered a risk factor for the development of hypertension, cardiovascular disease, diabetes mellitus, and several malignancies,^6^ which are potentially life‐threatening. However, all causes of death in obese patients were cancer‐related in the present study. Cancer progression might contribute to the poor prognosis in obese patients. Of note, however, Hassan et al reported that obesity did not affect the overall survival of patients with HCC despite an increased risk of HCC development in obese patients.^27^ In the present study, only obesity was identified as a significant risk factor for death. However, given the small number of deceased patients, the accumulation of more patients and a further investigation will be necessary to confirm the prognostic factors for overall survival.

Improvement of obesity theoretically can be related to less recurrence or better overall survival. However, in the present study, only four patients showed improvement of obesity. Therefore, further investigation is necessary to clarify the impact of improvement of obesity on recurrence or death.

Several limitations associated with the present study warrant mention. First, this study had a retrospective design and enrolled a small number of patients who achieved an SVR. Second, the current results may have been influenced by the long study duration, as medical techniques and treatment approaches may have changed over time. However, the long observational period might have clinical significance in the treatment of SVR patients with HCC. Third, because the treatment of HCV patients with direct‐acting antiviral agents (DAA) is now the standard of care, the findings might not fully apply to future HCV patients. However, the beneficial effects of DAA on HCC development or recurrence have not been determined.^28^, ^29^ This study may be useful as a reference for further investigations of HCC recurrence after achieving SVR induced by DAA.

In conclusion, obesity and non‐anatomical resection were independent risk factors for HCC recurrence after liver resection following successful IFN therapy for chronic hepatitis C. Obesity is an important clinical problem to consider in order to improve postoperative outcomes in such patients.

## DISCLOSURE

Conflicts of Interest: Authors declare no conflicts of interest for this article.
