# Supplementary material for: Obesity and recurrence‐free survival in patients with hepatocellular carcinoma after achieving sustained virological response to interferon therapy for chronic hepatitis C
Source: Ann Gastroenterol Surg. 2018 Jun 22;2(4):319–26. doi: 10.1002/ags3.12183 (PMC6036378; doi:10.1002/ags3.12183)
Supplement: Supplementary file 1 [file AGS3-2-319-s001.docx]

Supplementary Table 1. Characteristics of patients with HCC after HCV eradication

| Variable | SVR patients (n = 59) |
| --- | --- |
| Sex (male/female) | 48/11 |
| Age (years) ^a^ | 66 (63–72) |
| Body mass index (kg/m^2^) ^a^ | 23.9 (18.7–32.5) |
| Alcohol abuse (+/−) | 13/46 |
| Diabetes mellitus (+/−) | 17/42 |
| Dyslipidemia (+/−) | 12/47 |
| Hypertension (+/−) | 26/33 |
| Total bilirubin (mg/dL) ^b^ | 0.7 (0.5–0.9) |
| Albumin (g/dL) ^b^ | 4.2 (4.0–4.5) |
| Platelet count (×10^4^/mL) ^b^ | 15.6 (13.1–18.3) |
| ALT (IU/l) ^b^ | 26 (19–34) |
| α-fetoprotein (ng/ml) | 17 (35%) |
| >20 | 17 |
| ≤20 | 42 |
| ICG-R15 (%)^a^ | 13.7(2.0-38) |
| HBc antibody positive | 33(55.9%) |
| Interval from IFN* (months) ^b^ | 42(27-81) |
| HCV strains |  |
| HCV genotype (n = 36) |  |
| 1b | 26 |
| 2a | 7 |
| 2b | 2 |
| 2a+2b | 1 |
| HCV serotype (n = 21) |  |
| Group 1 | 12 |
| Group 2 | 9 |
| Unknown | 2 |
| Tumor size (cm) ^b^ | 2.0 (1.6–2.6) |
| Differentiation degree (well/mod/poor)^*^ | 5/40/14 |
| Tumor number (single/multiple) | 50/9 |
| Microvascular invasion (+/−) | 18/41 |
| Hepatic steatosis |  |
| (Score 0/1/2/3) | 27/17/11/4 |
| Lobular inflammation |  |
| (Score 0/1/2/3) | 2/10/36/11 |
| Ballooning  (Score 0/1/2) | 35/15/9 |
| Grading score for activity hepatitis |  |
| (Grade 0/1/2/3/4) | 2/33/23/1/0 |
| Liver cirrhosis (+/−) | 15/44 |
| Anatomic resection (+/−) | 22/37 |

ALT, alanine aminotransferase; HCV, hepatitis C virus; ICG-R15, indocyanine green retention rate at 15 minutes; ^*^Interval from the end of interferon therapy to the detection of hepatocellular carcinoma; ^#^ Tumor differentiation: well, well-differentiated; mod, moderately differentiated; poor, poorly differentiated; ^a^ median with range, ^b^ median with interquartile range
