# Supplementary material for: Obesity and recurrence‐free survival in patients with hepatocellular carcinoma after achieving sustained virological response to interferon therapy for chronic hepatitis C
Source: Ann Gastroenterol Surg. 2018 Jun 22;2(4):319–26. doi: 10.1002/ags3.12183 (PMC6036378; doi:10.1002/ags3.12183)
Supplement: Supplementary file 2 [file AGS3-2-319-s002.docx]

Supplementary Table 2. Overall survival after surgery

| Variables | No. | MST | Survival rate (years) | | | *P* |
| --- | --- | --- | --- | --- | --- | --- |
|  |  | (days) | 3 | 5 | 7 |  |
| Age (years) |  |  |  |  |  |  |
| < 65 | 21 | NA | 100 | 93 | 83 | 0.20 |
| ≥ 65 | 38 | 4006 | 100 | 85 | 71 |  |
| Gender |  |  |  |  |  |  |
| Female | 11 | NA | 100 | 100 | 100 | 0.44 |
| Male | 48 | 1339 | 100 | 86 | 71 |  |
| Alcohol abuse |  |  |  |  |  |  |
| Presence | 13 | 2662 | 100 | 83 | 63 | 0.60 |
| Absence | 46 | 5838 | 100 | 90 | 80 |  |
| Diabetes mellitus |  |  |  |  |  |  |
| Presence | 17 | NA | 100 | 89 | 59 | 0.69 |
| Absence | 42 | 5838 | 100 | 89 | 75 |  |
| Obesity |  |  |  |  |  |  |
| Presence | 18 | 2617 | 100 | 80 | 64 | 0.014 |
| Absence | 41 | 5838 | 100 | 92 | 82 |  |
| Dyslipidemia |  |  |  |  |  |  |
| Presence | 12 | NA | 100 | 100 | 100 | 0.17 |
| Absence | 47 | 5838 | 100 | 87 | 73 |  |
| Hypertension |  |  |  |  |  |  |
| Presence | 26 | 2617 | 100 | 84 | 61 | 0.32 |
| Absence | 33 | 5838 | 100 | 91 | 85 |  |
| HBc antibody |  |  |  |  |  |  |
| Positive | 33 | NA | 100 | 90 | 75 | 0.61 |
| Negative | 26 | 5838 | 100 | 87 | 78 |  |
| Interval from IFN* (years) |  |  |  |  |  |  |
| ≤5 | 39 | 5838 | 100 | 84 | 72 | 0.71 |
| >5 | 20 | 4006 | 100 | 100 | 88 |  |
| T-Bil (mg/dL) |  |  |  |  |  |  |
| <1.0 | 48 | NA | 100 | 90 | 79 | 0.75 |
| ≥1.0 | 11 | 5838 | 100 | 86 | 72 |  |
| Albumin (g/dL) |  |  |  |  |  |  |
| <4.0 | 14 | 2662 | 100 | 89 | 59 | 0.32 |
| ≥4.0 | 45 | 5838 | 100 | 89 | 83 |  |
| Platelet count (×10^4^/mL) |  |  |  |  |  |  |
| <15 | 29 | 2662 | 100 | 90 | 68 | 0.23 |
| ≥15 | 30 | 5838 | 100 | 87 | 87 |  |
| ALT (IU/l) |  |  |  |  |  |  |
| >30 | 21 | 5838 | 100 | 85 | 75 | 0.76 |
| ≤30 | 38 | NA | 100 | 92 | 78 |  |
| α-fetoprotein (ng/ml) |  |  |  |  |  |  |
| >20 | 17 | 5838 | 100 | 75 | 66 | 0.28 |
| ≤20 | 42 | 4006 | 100 | 96 | 81 |  |
| Tumor size (cm) |  |  |  |  |  |  |
| >2.0 | 29 | NA | 100 | 94 | 81 | 0.37 |
| ≤2.0 | 30 | 4006 | 94 | 83 | 71 |  |
| Diff. degree^#^ |  |  |  |  |  |  |
| Well, mod | 45 | 5838 | 100 | 96 | 82 | 0.17 |
| Por | 14 | NA | 100 | 75 | 64 |  |
| Tumor number |  |  |  |  |  |  |
| Single | 50 | 5838 | 100 | 90 | 80 | 0.25 |
| Multiple | 9 | 2105 | 100 | 83 | 42 |  |
| MVI |  |  |  |  |  |  |
| Presence | 18 | NA | 100 | 100 | 100 | 0.18 |
| Absence | 41 | 4006 | 100 | 89 | 75 |  |
| Hepatic steatosis |  |  |  |  |  |  |
| Positive | 32 | 2617 | 100 | 84 | 59 | 0.10 |
| Negative | 27 | 5838 | 100 | 94 | 94 |  |
| Lobular inflammation |  |  |  |  |  |  |
| score = 2/3 | 47 | 5838 | 100 | 86 | 76 | 0.32 |
| score = 0/1 | 12 | 4006 | 100 | 100 | 100 |  |
| Balooning |  |  |  |  |  |  |
| Positive | 24 | 5838 | 100 | 84 | 63 | 0.26 |
| Negative | 35 | NA | 100 | 92 | 86 |  |
| Liver cirrhosis |  |  |  |  |  |  |
| Presence | 15 | 2662 | 100 | 83 | 56 | 0.19 |
| Absence | 44 | 5838 | 100 | 92 | 86 |  |
| Type of hepatic resection |  |  |  |  |  |  |
| Non-anatomic | 37 | NA | 100 | 83 | 72 | 0.66 |
| Anatomic | 22 | 5838 | 100 | 87 | 79 |  |

ALT, alanine aminotransferase; MST, median survival time; MVI, microvascular invasion; ^*^Interval from the end of interferon therapy to the detection of hepatocellular carcinoma; ^#^Tumor differentiation: por, poor; mod, moderate.
